# Application of Artificial Intelligence Methods on Osteoporosis Classification with Radiographs—A Systematic Review

**DOI:** 10.3390/bioengineering11050484

**Published:** 2024-05-12

**Authors:** Ren Wei Liu, Wilson Ong, Andrew Makmur, Naresh Kumar, Xi Zhen Low, Ge Shuliang, Tan Yi Liang, Dominic Fong Kuan Ting, Jiong Hao Tan, James Thomas Patrick Decourcy Hallinan

**Affiliations:** 1 Department of Diagnostic Imaging, National University Hospital, 5 Lower Kent Ridge Road, Singapore 119074, Singaporedominic.fong@mohh.com.sg (D.F.K.T.);; 2 Department of Diagnostic Radiology, Yong Loo Lin School of Medicine, National University of Singapore, 10 Medical Drive, Singapore 117597, Singapore; 3 University Spine Centre, Department of Orthopaedic Surgery, National University Health System, 1E, Lower Kent Ridge Road, Singapore 119228, Singapore

**Keywords:** artificial intelligence, machine learning, deep learning, osteoporosis, imaging, radiographs

## Abstract

**Simple Summary:**

Osteoporosis is a major global health problem with substantial economic and psychosocial repercussions. Underdiagnosis of osteoporosis is prevalent. The dual-energy absorptiometry (DEXA) scan is the gold standard for bone mineral density (BMD) measurement but its accessibility is limited. Radiographs are ubiquitous in healthcare and represent a promising avenue for opportunistic osteoporosis screening. Historically, this has been associated with high labor and time costs but several recent studies have demonstrated that BMD can be estimated from radiographs in a cost-effective manner utilizing deep learning techniques. This review aims to summarize the existing evidence supporting the utility of artificial intelligence (AI) methods for osteoporosis classification using radiographs.

**Abstract:**

Osteoporosis is a complex endocrine disease characterized by a decline in bone mass and microstructural integrity. It constitutes a major global health problem. Recent progress in the field of artificial intelligence (AI) has opened new avenues for the effective diagnosis of osteoporosis via radiographs. This review investigates the application of AI classification of osteoporosis in radiographs. A comprehensive exploration of electronic repositories (ClinicalTrials.gov, Web of Science, PubMed, MEDLINE) was carried out in adherence to the Preferred Reporting Items for Systematic Reviews and Meta-Analyses 2020 statement (PRISMA). A collection of 31 articles was extracted from these repositories and their significant outcomes were consolidated and outlined. This encompassed insights into anatomical regions, the specific machine learning methods employed, the effectiveness in predicting BMD, and categorizing osteoporosis. Through analyzing the respective studies, we evaluated the effectiveness and limitations of AI osteoporosis classification in radiographs. The pooled reported accuracy, sensitivity, and specificity of osteoporosis classification ranges from 66.1% to 97.9%, 67.4% to 100.0%, and 60.0% to 97.5% respectively. This review underscores the potential of AI osteoporosis classification and offers valuable insights for future research endeavors, which should focus on addressing the challenges in technical and clinical integration to facilitate practical implementation of this technology.

## 1. Introduction

### 1.1. Osteoporosis—A Global Health Challenge

Osteoporosis is a complex endocrine disorder affecting bone turnover, marked by a decline in bone mass and microstructural integrity [1]. Although asymptomatic in the early stages, it significantly elevates the risk of fragility fractures, leading to increased morbidity, mortality, and reduced quality of life [2].

The disease constitutes a major global health problem [3,4], affecting one-third of women and one-fifth of men aged 50 and above [5,6,7]. Worldwide, an estimated 200 million women suffer from osteoporosis [8], a figure that is projected to rise with the aging global population and increasing life expectancy. The economic and psychosocial repercussions of fragility fractures are enormous [9,10,11,12], with a 2005 United States study reporting direct costs exceeding USD 17 billion [13] and a 2013 European Union study estimating a total economic burden of EUR 37 billion and loss of 1,180,000 quality-adjusted life years in 2010 [14].

Although cost-effective treatments are readily available [15], underdiagnosis and undertreatment remain prevalent. Importantly, out of female patients suffering their first osteoporotic fracture, only an estimated 10.3% will have had undergone prior bone mineral density (BMD) testing [14,16,17,18].

### 1.2. Current Diagnostic Methods and Challenges

Dual-energy absorptiometry scans (DEXA) of the lumbar spine and hip, advocated by the World Health Organisation (WHO) since 1987, is a gold standard in BMD measurement. DEXA is non-invasive and cost-effective and remains the most frequently utilized radiologic technique for evaluating bone mass [8,19,20]. It is also validated for use with other tools such as the WHO fracture risk assessment (FRAX) algorithm [20]. However, DEXA has its downsides including limited accessibility, need for strict quality standards, operator dependency [21], and suboptimal screening rates [12,18]. Alternative techniques for measuring BMD such as quantitative computed tomography [22], ultrasound [23], and peripheral DEXA [24] are yet to see widespread clinical use.

### 1.3. Potential of Machine Learning for Osteoporosis Classification

Radiographs are the most frequently utilized imaging modality in healthcare. The potential for osteoporosis classification via radiographs has been explored since the 1960s. For example, Barnett et al. measured lumbar vertebral concavity along with the femoral and metacarpal cortical thicknesses by hand with a millimeter ruler as an osteoporosis scoring procedure [25,26]. In addition, Garn et al. documented various techniques in the use of densitometry to diagnose osteoporosis [27], including the simultaneous use of metallic phantoms in radiography [28,29]. These initial manual methods were associated with significant manual labor costs and exacting technical processing requirements, limiting the feasibility of large-scale use.

Machine learning, a subset of AI, holds great promise for the automated segmentation and classification of large volumes of medical imaging data. Recent studies employing modern convolutional neural networks and deep learning architectures have demonstrated that BMD can be accurately estimated from radiographs in a cost-effective manner. This review aims to summarize the existing evidence supporting the utility of AI methods for osteoporosis classification using radiographs.

### 1.4. Research Questions

This review aims to synthesize existing evidence supporting the utility of AI methods for osteoporosis classification using radiographs. Key research questions include:

1. How effective are AI methods in accurately classifying osteoporosis using radiographic data?
2. What are the current technical challenges and practical limitations in osteoporosis diagnosis and classification?
3. What are potential future directions for the use of AI-based classification in osteoporosis management?

## 2. Materials and Methods

### 2.1. Literature Review

A query of prominent electronic databases (clinicaltrials.gov, Web of Science, MEDLINE, PubMed) was performed in alignment with the Preferred Reporting Items for Systematic Reviews and Meta-Analyses 2020 statement (PRISMA). The search utilized the following specific search terms: (“neural network*” OR “convolutional neural network*” OR “machine learning” OR “deep learning” OR “AI” OR “artificial intelligence”) AND ((“bone” AND “mineral” AND “density”) OR “BMD”) OR “osteoporosis” OR “absorptiometry” OR “DEXA” OR “osteopaenia” OR “osteopenia” AND (“X-ray” OR (“Radiographs”)). Two reviewers (R.L. and W.O.) screened the resultant articles and shortlisted a number for further examination. The studies were then reviewed and discussed to reach a consensus on their suitability before inclusion. Any disagreements were resolved by a third reviewer (J.T.P.D.H.). The database query was limited to articles published prior to 14 August 2023.

### 2.2. Screening of Studies and Criteria for Selection

No constraints were specified for the literature and reference search. Articles pertaining to deep learning, artificial intelligence (AI), or deep learning to categorize or predict osteoporosis from radiographs were shortlisted, including those with comparative analysis to conventional DEXA scans when available.

### 2.3. Extraction of Data and Reporting

Using Microsoft Excel (Microsoft Corporation, Washington, DC, USA), a list of selected papers was compiled. The compiled information encompassed the following:

- Main clinical use: Classify osteoporosis through the application of machine learning tools (e.g., BMD estimation or bi-variate/tri-variate classification, i.e., normal, osteopenic, and osteoporotic);
- Research article characteristics: Comprehensive authorship, publication date, and journal or publication name, all written in the English language;
- Research protocol: Study design, anatomical region, imaging modality, patient demographics, and healthcare context;
- Machine Learning: Type of machine learning architecture or technique used, need for human supervision or pre-processing.

## 3. Results

### 3.1. Search Results

**Figure 1:**
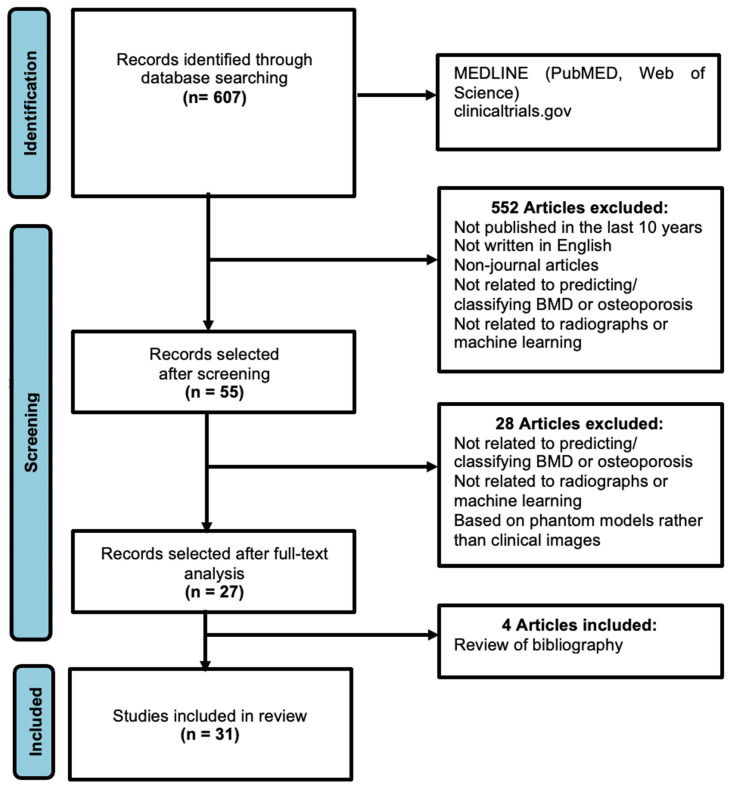
PRISMA flowchart for the literature search (adapted from the PRISMA group, 2020), which describes the selection of relevant articles.

The initial literature review (Figure 1) yielded 607 relevant research articles, which were then screened using the aforementioned criteria. Following the screening, 55 articles were selected, which were further analyzed to determine suitability by two readers. Any discordance was resolved after discussion with a third reader. Upon review, a further 28 publications were excluded as they did not involve AI analysis of radiographs or relied on phantom models rather than patient data. In addition, four articles were added after reviewing the references of the chosen articles. Overall, this resulted in a total of 31 articles (Table 1) for in-depth analysis. The main findings were consolidated and summarized in this systematic review. Due to insufficient data in most studies, formal meta-analysis using 2 × 2 contingency tables was not feasible.

### 3.2. Model Accuracy in Classification of Osteoporosis

The pooled reported accuracy, sensitivity, and specificity of AI osteoporosis classification ranges from 0.661 to 0.9787, from 0.674 to 1, and from 0.60 to 0.9751, respectively, with AUC values of 0.70 to 0.9987. Most papers used DEXA as the benchmark for comparison. One study assessing knee radiographs used quantitative ultrasound as a reference standard [30] and another examined machine versus human osteoporosis classification based on second metacarpal cortical percentage [31].

Out of the 31 studies, one study examined both hip and spine radiographs, while the other 30 studies examined different anatomical regions as detailed in Table 2. Deep learning models in all anatomical areas showed overall good AUC and accuracy; however, studies on calcaneal and dental radiographs generally yielded higher AUC and accuracy relative to other anatomical regions. For example, Singh et al. performed a study assessing calcaneal radiographs for osteoporosis and noted that the relative paucity of soft tissue around the calcaneum was advantageous, as soft tissue could increase measurement variability. Their study yielded an excellent AUC of 0.9824 and an accuracy of 0.9787 using a support vector machine classifier [32]. In contrast, Cui et al. [33] described the negative impact of soft tissue, bowel gas, and clothing artefacts in their analysis of lumbar spine radiographs and made use of various image processing algorithms to mitigate the problem.

In their respective studies analyzing chest radiographs, Jang et al. [34] noted that it was unclear how incidental findings such as calcified nodules or old fractures may confound osteoporosis classification, while Sato et al. [35] suggested that it may be worthwhile for models to be supplied with clinical information on relevant comorbidities such as fracture history, chronic obstructive pulmonary disease, and rheumatoid arthritis to improve accuracy. The region of assessment can therefore have a significant impact on performance; a model must incorporate various techniques to remain robust in the analysis of different anatomical regions in the clinical setting.

### 3.3. Study Protocol and Performance Metrics

There is variation in the study protocols and performance metrics used to report model efficacy. For example, some studies performed a binary classification distinguishing osteoporosis and non-osteoporosis and hence relied on metrics such as accuracy. Other authors predicted BMD values for each patient and compared these against the gold standard of DEXA using the correlation co-efficient R. A few of the different performance metrics used are briefly explained below:

1. **Accuracy:** Accuracy represents the proportion of correctly classified instances among all instances examined. It provides a general measure of model performance but may not be suitable for imbalanced datasets;
2. **Sensitivity and Specificity:** Sensitivity (true positive rate) measures the proportion of actual positives that are correctly identified by the model, while specificity (true negative rate) measures the proportion of actual negatives that are correctly identified by the model;
3. **Area Under the Curve (AUC):** AUC refers to the area under the receiver operating characteristic (ROC) curve, which illustrates the trade-off between sensitivity and specificity across various threshold settings. A higher AUC value indicates better discrimination ability of the model;
4. **F1 Score:** The F1 score is the harmonic mean of precision and recall (sensitivity). It provides a balance between precision (the proportion of true positive predictions among all positive predictions) and recall, making it suitable for imbalanced datasets;
5. **Correlation Coefficient (R):** The correlation coefficient measures the strength and direction of the linear relationship between two variables. In the context of osteoporosis classification, it reflects the agreement between predicted bone mineral density values (a scalar numerical value) and gold standard measurements obtained from DEXA scans.

### 3.4. Machine Learning in Medical Imaging

Artificial intelligence is defined in the Merriam-Webster dictionary as “the capability of computer systems or algorithms to imitate intelligent human behaviour”. Machine learning, a subset of artificial intelligence, employs algorithms and statistical techniques to allow computer systems to learn and make informed predictions from data. Several systems have demonstrated diagnostic capabilities comparable to medical professionals in various clinical conditions [36], with many software applications being approved for clinical use [37,38]. Various diagnostic imaging models are available for chest radiographs [39], mammograms [40], and MRI spine analysis [41], with the last showing improved productivity for the reporting radiologist [42]. Radiomics, which refers to the quantitative extraction of various characteristics from a medical image in order to facilitate statistical analysis [43,44], has also been augmented by the use of artificial intelligence.

**Figure 2:**
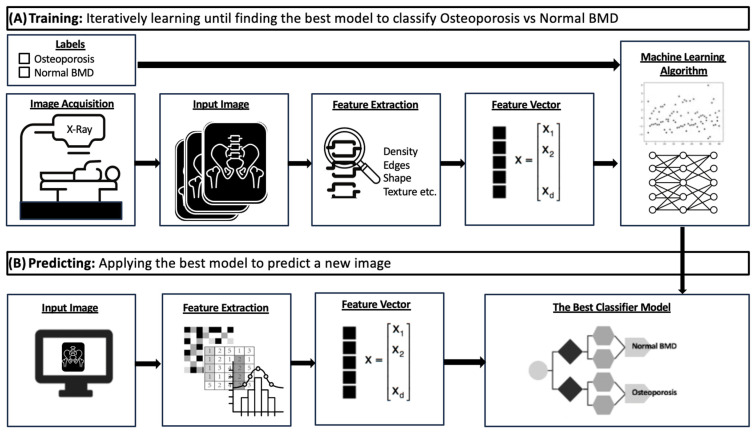
Diagram showing model development and application in the classification of medical images. The top row depicts the training process (**A**) and the bottom row the prediction process (**B**).

Figure 2 outlines a general procedure for the development, testing, and deployment of a machine-learning model [45,46,47]. A dataset of medical images would first be collected and pre-processed manually or via automated methods. An appropriate model is then selected and trained using a portion of the dataset (the training set), adjusting internal parameters to improve prediction accuracy. After training, the model is tested on a separate unseen dataset (test set) and fine-tuned to optimize its performance and minimize error.

1. **Data Collection:** Usually, approval from an ethics committee is necessary before utilizing medical data for the development of a commercial or research AI algorithm. In the case of a prospective study, explicit informed consent is necessary. Medical imaging data are usually collated from a picture archiving and communication system (PACS) environment, requiring collaboration between AI developers and healthcare professionals [48]. Accessing relevant data involves querying, appropriately de-identifying, and securely storing the information. Protected health information must be removed from both the Digital Imaging and Communications in Medicine (DICOM) metadata and the images themselves [49];
2. **Image Processing and Segmentation:** Segmentation is the process of delineating structures within a medical image, thereby creating structured visual representations from unstructured raw data [47,50]. For example, in tumor segmentation, this could be the process of defining the margins of a tumor [45]. For osteoporosis classification, this could refer to the separation of bone and non-bone structures [51,52];
3. **Training and Validation:** An appropriate model is selected and trained using a portion of the dataset. Supervised machine learning models are provided with data inputs labeled by human experts, whereas unsupervised models extract salient features from unlabeled data to uncover meaningful relationships within datasets. Models determine how to perform meaningful feature extraction and computation, which involves the evaluation of the image factors that allow a prediction to be made. Image features should be independent of technical factors such as noise, signal, and rotation, as these are common issues in medical images. Machine learning models typically iterate to improve performance with each exposure to the validation set;
4. **Testing:** The model is then tested on a set of examples to evaluate its diagnostic accuracy and performance [45]. Testing can be performed with internal and external test sets; the former refers to data that come from the same pool as the training set whereas the latter refers to data that have been collected from a different source. Good model performance on external testing sets bolsters confidence in model accuracy, whereas poor performance on external datasets may suggest overfitting [44,53].

Various machine-learning models can be used in medical imaging. In particular, convolutional neural networks (CNN) are a subset of machine learning models, which are frequently applied in medical imaging. CNNs utilize convolution kernels, which move across an input image to create a set of output values that are more suitable for analysis by a neural network [54,55]. Various CNN architectures, for example, LeNet, GoogleNet, AlexNet, VGGNet, and ResNet have shown efficacy in machine learning competitions, research, and clinical use. Open-source machine learning libraries such as TensorFlow, pyTorch, and Keras are available for public use, fostering widespread adoption of this technology.

**Table 1:** Selected articles; main characteristics.

Authors	Artificial Intelligence Method	Publication Year	Main Objectives	Title of Journal	Type of Radiographs	Performance
Ho, C.S. et al. [56]	CNN	2021	Classify osteoporosis	Arch. Osteoporos.	Pelvis and femur	*r* = 0.850; Accuracy 88.0%
Fathima, S.M.N. et al. [57]	CNN (U-Net)	2020	Classify osteoporosis	J Xray Sci Technol.	Various	Accuracy 88.0%; Sensitivity 95.2–95.8%; Specificity 96.7–97.5%
Hsieh, C.I. et al. [58]	CNN (VGG-16 and ResNet-34)	2021	Classify osteoporosis	Nat. Commun.	Lumbar spine, pelvis	AUC 0.890; Accuracy 86.2–91.7%; Sensitivity 80.2%–83.5%; Specificity 88.3%–94.9%
Sukegawa, S. et al. [59]	CNN (EfficientNet-b0, -b3, and -b7 and ResNet-18, -50, and -152)	2022	Classify osteoporosis	Sci. Rep.	Dental panoramic	AUC 0.911–0.921; Accuracy 84.0–84.5%; Specificity 88.8–90.6%; F1 score 0.720–0.740
Yamamoto, N. et al. [60]	CNN (ResNet18, ResNet34, GoogleNet, EfficientNet b3, EfficientNetb4)	2020	Classify osteoporosis	Biomolecules	Hip	Accuracy 88.5%; Specificity 92.2%; Recall 0.887; F1 score 0.894; AUC 0.922–0.937
Wani, I. et al. [30]	CNN (AlexNet, VggNet-16, ResNet, VggNet-19)	2022	Classify osteoporosis	Multimed. Tools Appl.	Knee	Accuracy 90.9%; Error rate 9.0%; Validation loss 54%
Lee, K.S. et al. [61]	CNN (CNN3, VGG-16, VGG-16_TF, VGG-16_TF_FT)	2020	Predict osteoporosis	J Clin Med.	Dental panoramic	AUC 0.858; Sensitivity 90.0%; Specificity 81.5%; Accuracy 84.0%
Zhang, B. et al. [62]	CNN	2020	Classify osteoporosis	Bone	Lumbar spine	AUC 0.767–0.810; Sensitivity 68.4–85.3%
Singh, A. et al. [32]	SVM, GNB, k-NN, ANN	2017	Predict osteoporosis	Comput Biol Med.	Calcaneum	AUC 0.982; Accuracy 97.9%; Sensitivity 100.0%; Specificity 95.7%
Tecle, N. et al. [31]	CNN (FSN-8)	2020	Predict osteoporosis	J Hand Surg Am.	Hand	Sensitivity 82.4%; Specificity 94.3%
Areeckal, A. S. et al. [63]	k-NN	2017	Predict osteoporosis	Osteoporos Int.	Hand and wrist	Accuracy 93.2%; Sensitivity 91.2%; Specificity 95.0%
Kavitha, M.S. et al. [64]	naive Bayes, k-NN, SVM	2015	Predict osteoporosis	Oral Surg Oral Med Oral Pathol Oral Radiol.	Dental panoramic	Accuracy 89.5–96.8%
Kavitha, M.S. et al. [65]	hybrid GSF	2016	Classify osteoporosis	Dentomaxillofac Radiol.	Dental panoramic	AUC 0.986; Sensitivity 99.1%; Specificity 98.4%; Accuracy 98.9% (femoral neck); AUC 0.962; Sensitivity 95.3%; Specificity 94.7%; Accuracy 96.0% (lumbar spine)
Chu, P. et al. [66]	CNN (OSN; AlexNet)	2018	Predict osteoporosis	Annu Int Conf IEEE Eng Med Biol Soc	Dental panoramic	Accuracy 89.8%
Hwang, J.J. et al. [67]	decision tree, SVM	2017	Predict osteoporosis	Dentomaxillofac Radiol	Dental panoramic	Accuracy 96.2–96.3%; Sensitivity 97.1–97.2%; Specificity 96.3–97.1
Lee, J.S. et al. [68]	SC-DCNN, SC DNN Augment, MC-DCNN	2019	Predict osteoporosis	Dentomaxillofac Radiol	Dental panoramic	AUC 0.973–0.999; Accuracy 93.0–98.5%
Oulhaj. H. et al. [69]	SVM	2017	Predict osteoporosis	IEEE Trans Med Imaging	Calcaneum	AUC 0.930; Accuracy 91.3%; Sensitivity 92.0%; Specificity 91.0%
Zheng, K. et al. [70]	CNN (Alexnet, Googlenet, Resnet18, Inceptionv3)	2020	Predict osteoporosis	Artif Intell Med	Calcaneum	AUC 0.944; Accuracy 90.8%
Nasser, Y. et al. [71]	SVM	2017	Predict osteoporosis	New York: IEEE	Calcaneum	Accuracy 95.5%
Jang, M. et al. [34]	Deep learning model (OsPor-screen)	2022	Classify osteoporosis	J. Bone Miner. Res.	Chest	AUC 0.880–0.910; Accuracy 77.7–84.2%; Sensitivity 84.3–86.2%; Specificity 74.2–81.5%
Bhattacharya, S. et al. [72]	SVM, NN	2019	Classify osteoporosis	IEEE Xplore	Calcaneum	Accuracy 95.6%
Jang, R. et al. [73]	CNN (VGG16)	2021	Classify osteoporosis	Sci. Rep.	Hip	AUC 0.700; Accuracy 81.2%; Sensitivity 91.1%; Specificity 68.9%,
Nguyen, T. et al. [74]	CNN	2021	Classify osteoporosis	Comput. Biol. Med.	Hip	*r* = 0.808
Singh, Y. et al. [75]	CNN	2021	Classify osteoporosis	43rd Conf Proc IEEE Eng Med Biol Soc	Dental panoramic	Accuracy: 87.9%
Sato, Y. et al. [35]	CNN	2022	Classify osteoporosis	Biomedicines.	Chest	AUC 0.700–0.890; Accuracy 66.1–78.5%; Sensitivity 71.3–90.1%; Specificity 62.4–73.7%
Hong, N. et al. [76]	CNN	2023	Classify osteoporosis	J Bone Miner Res.	Lateral spine	AUC 0.830–0.850; Sensitivity 75.0–76.0%
Nakamoto, T. et al. [77]	CNN	2022	Classify osteoporosis	Dentomaxillofac Radiol	Dental panoramic	Sensitivity 78.3–82.6%; Specificity 71.4–79.2%; Accuracy 74.0–79.0% (Lumbar spine DEXA); Sensitivity 80.0–86.7%; Specificity 67.1–74.1%; Accuracy 70.0–75.0% (Femoral Neck)
Widyaningrum, R. et al. [51]	DT, GNB, MLP	2023	Classify osteoporosis	Int. J. Dent.	Dental panaromic	Accuracy 90.5%; Specificity 90.9%; Sensitivity 90.0%
Lee, S.W. et al. [78]	CNN	2020	Classify osteoporosis	Skeletal Radiol.	Spine	AUC 0.740; Accuracy 71.0%; Sensitivity 81.0%; Specificity 60.0%; F1-score 0.73
Mohammadi, F. G. et al. [79]	CNN	2023	Classify osteoporosis	Stud Health Technol Inform	Hand	AUC 0.740; Accuracy 82.0%; Sensitivity 87.0%; Specificity 61.0%
Mao, L. et al. [80]	CNN	2022	Classify osteoporosis	Front. Endocrinol.	Lumbar spine	AUC 0.937; Sensitivity 84.8%; Specificity 86.6%

Area under receiver operator curve (AUC), correlation coefficient (r).

**Table 2:** Results summary.

Areas Sampled	No. of Studies	AUC	Accuracy (%)	Sensitivity (%)	Specificity (%)
Dental	10	0.858–0.999	74.0–96.9	78.3–97.2	67.1–97.1
Hip	5	0.700–0.937	81.2–88.5	80.2–91.1	68.9–94.9
Spine	5	0.726–0.937	71.0–86.2	68.4–84.8	60.0–88.3
Calcaneum	5	0.930–0.982	90.8–97.9	92.0–100.0	91.0–95.7
Hand or Wrist	3	0.740	82.0–93.2	82.4–91.2	61.0–95.7
Chest	2	0.700–0.910	66.1–84.2	71.3–90.1	62.4–81.5
Various *	1	-	88.0	95.2–95.8	96.7–97.5
Knee	1	-	90.9	-	-
Overall	32	0.700–0.999	66.1–97.9	67.4–100.0	60.0–97.5

* Internal datasets were used consisting of spine, femur, knee, clavicle, and upper extremity radiographs.

## 4. Discussion

### 4.1. Advantages and Efficacy

Based on our comprehensive review, many machine-learning tools demonstrate impressive diagnostic capabilities for osteoporosis when benchmarked against established reference standards. Furthermore, these tools consistently show excellent discriminatory performance across various anatomical regions, with promising outcomes in osteoporosis prediction using radiographs of the hip, spine, chest, extremities, and mandible.

One major advantage is manpower and time savings for image segmentation and analysis. Segmentation is the process of identifying regions of interest (ROI) in images, such as separating bone and non-bone structures on radiographs. Historically, this was a manual and time-consuming task requiring trained personnel. In contrast, AI can handle vast imaging datasets without manual intervention, reducing human error and interobserver variability. As a case in point, Jang et al. noted that their automated “OsPor-screen” model required less than 4 s to process and classify a chest radiograph [34]. Similarly, Doctorant et al. described an AI model for ROI labeling in lumbar spine DEXA, which required only seconds for analysis and matched the performance of expert operators [52].

In addition, radiographs are an ideal modality for large-scale population screening due to their cost-effectiveness and typically lower radiation exposure when compared to DEXA and quantitative CT scans. Existing radiographs performed for other purposes may also be retrospectively analyzed for osteoporosis without incurring further costs or radiation burden to the patient, increasing the appeal of screening and improving screening program uptake in the population. Serial radiographs may also be performed for a patient over time depending on the clinical scenario, such as follow-up of chest infections and assessing fracture healing. This would permit the close trending of BMD without the need for frequent DEXA scans, although the clinical utility of this process is currently uncertain and requires further evaluation.

Finally, radiographs represent a promising alternative avenue for osteoporosis diagnosis in rural settings or developing countries where DEXA machines are not widely available, a role that is analogous to that of quantitative ultrasounds [81,82]. Given the high diagnostic accuracy of some deep learning models (AUCs up to 0.9987 in dental [68] and 0.9824 in calcaneal radiographs [32]), further research and validation is merited to establish if deep learning diagnosis of osteoporosis from radiographs in tandem with other clinical tools may suffice for treatment initiation in these underserved populations.

### 4.2. Challenges: Dataset Collection

**Figure 3:**
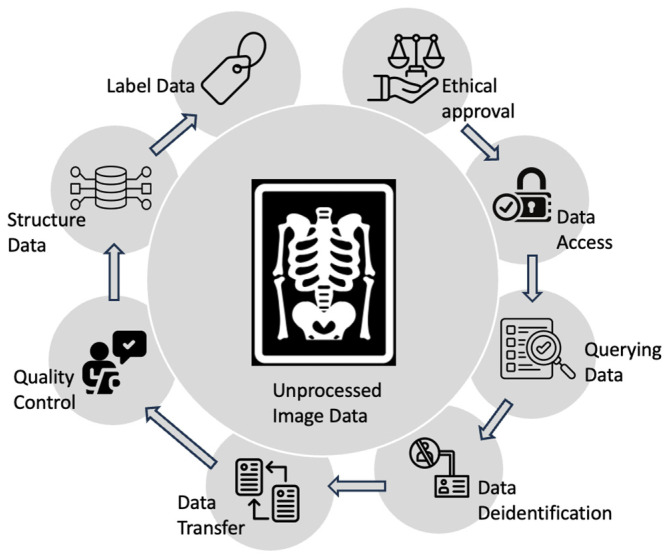
Diagram showing the process of medical image data handling for machine learning.

The efficacy of a machine learning model is contingent on the size and quality of the dataset on which it is trained [48,49]. There are several challenges in medical image collection (Figure 3); first, data collection is subject to ethical considerations such as patient privacy and radiation exposure. Second, medical images can be very large in size with high resolutions. Finally, imaging platforms may not be readily amenable to data transfer and collection in view of security concerns [83]. Therefore, medical imaging datasets tend to be relatively small, comprising hundreds to thousands of images [48] compared to natural image datasets, which can contain millions of images.

Deep learning models trained on small datasets tend to have reduced generalizability and overfitting. To mitigate this problem, data augmentation techniques (such as blurring, shearing, sharpening, and rotation) are often performed to artificially increase the size of datasets without the need for further collection [84]. Transfer learning is also a relevant technique, whereby a CNN trained on a large dataset may be retrained on a smaller dataset for a new problem [30,85]. For example, Wani et al. [30] and Lee et al. [61] independently utilized transfer learning in the analysis of knee and dental radiographs, respectively, noting that the use of a pre-trained model helps to mitigate the problem of smaller datasets.

Dataset bias occurs when a dataset used to train a model has a different distribution from the population to which it is to be applied [86,87]. Some studies only examine a small subgroup of the population and thus may appear accurate if the test sets are derived from the same population but fail when tested in a broader context. A recent review of studies analyzing dental radiographs by Martins et al. [88] noted that most papers only incorporated data from a single institute, while Alberquerque et al. [89] noted the problem of dataset imbalance, whereby a dataset retrospectively including patients who had previously undergone DEXA scans would be skewed toward higher rates of osteoporosis than the general population. It can be difficult to determine if a dataset is subject to bias, particularly if the collection criteria are not disclosed. Critical evaluation of datasets by researchers and meticulous documentation of a dataset’s characteristics and patient demographics would help to alleviate this problem [83].

### 4.3. Challenges: Radiograph Quality and Confounding Pathologies

Another significant challenge lies in the technical quality of radiographs, as suboptimal positioning, variable imaging techniques, variations in image exposure, and the presence of artifacts can lead to inaccuracies in the model. One study by Socha et al. [90] examining COVID-19 detection AI models described how poor image quality, artifacts, and data heterogeneity in the initial datasets collected during the pandemic contributed to poor performance in real-world clinical settings. In the context of osteoporosis, Hsieh et al. [58] noted that bony pathologies such as fractures, implants, bony tumors, infections, and severe osteoarthritis can introduce complexity in analysis. It was postulated that fractures can alter normal bone anatomy and induce callus formation, whereas implants might produce metallic artifacts, rendering the evaluation of adjacent tissue more challenging. The presence of these factors necessitates algorithms and image processing methods to differentiate true BMD alterations from other non-BMD changes. Fortunately, the ubiquity of radiographs may partially circumvent this problem; for example, in a large AI pipeline, a patient with a hip implant may have radiographs of other anatomical regions such as the chest and other limbs performed at the same sitting and thereby undergo osteoporosis screening even though the hip radiograph may not be amenable to analysis [58].

The advent of AI tools for the detection of other bone pathologies may also help to mitigate the problem. For example, Hsieh et al. [58] incorporated multiple deep learning models in their automated osteoporosis detection pipeline, such as for the detection of fractures and other algorithms for the exclusion of poor-quality images and concomitant pathologies [58]. The tool was able to automatically exclude confounding radiographs and successfully report a predicted BMD in 79.0% of pelvis radiographs and 82.3% of spine radiographs in a large population of tertiary care patients at a general hospital without manual intervention.

### 4.4. Challenges: Study Protocol Heterogeneity

The variability in study protocols presents significant challenges in the field of AI-driven osteoporosis classification. Differences in the choice of inclusion/exclusion criteria, pre-processing methodology, and model construction make it difficult to compare findings across studies.

Moreover, inconsistencies in defining model endpoints and the choice of performance metrics further complicate the interpretation of results. For example, some papers opt to classify osteoporosis versus non-osteoporosis in patients while others endeavor to measure BMD directly. This diversity underscores the complexity of the issue and highlights the importance of standardizing methodologies to ensure consistency and reliability in research outcomes. Improved consistency in study protocols and standardizing methodologies will facilitate the accumulation of robust evidence, ultimately advancing our understanding of AI-driven solutions.

### 4.5. Challenges: Clinical Integration

The clinical integration of artificial intelligence solutions is fraught with challenges [91]. Recht et al. [92] outlined various ethical, technical and clinical challenges involved in the clinical integration of AI: AI algorithms must align with the complex and diverse spectrum of clinical protocols and practices across different regions and healthcare contexts. Data privacy and security concerns, along with the ethical implications of AI-driven decision support, also add layers of complexity to the integration process. Daye et al. [93] proposed a roadmap for successful oversight of clinical AI implementation, noting that four components are required for successful implementation: data access and security, cross-platform and cross-domain integration, clinical translation and delivery, and leadership supporting innovation.

Furthermore, the regulatory landscape is continuously evolving, necessitating frequent updates and adaptations to ensure AI systems comply with rigorous healthcare safety and quality standards [94]. Close cooperation with regulatory bodies such as the Food and Drug Administration in the United States is required [95].

Variability in data formats, quality, and acquisition techniques across various healthcare systems can also hinder the performance of AI tools. The heterogeneous distribution of disease in various populations and different populations also further complicates matters and may necessitate the use of separate training sets in different populations [90]. Most of the reviewed studies demonstrate good diagnostic accuracy on internal or external datasets without the inclusion of an integrated clinical pathway. On the other hand, Hsieh et al. [58]. outlined a process at the Chang Gung Memorial Hospital (Linkou, Taiwan) in which the hospital PACS relayed all newly acquired pelvic and lumbar spine radiographs to an inference platform daily [58]. The system also integrated several other deep learning tools for the detection of image quality and other bony pathologies such as hip or lumbar spine fractures and automatically excluded these studies. There is a clear need for further research and infrastructure development to support the establishment of similar integrated clinical platforms.

As a counterpoint, various studies do make use of clinical data to improve diagnostic accuracy. For example, Yamato et al. [60], Sukegawa et al. [59], and Mao et al. [80] describe the use of ensemble models incorporating clinical covariate data such as age, gender, and BMI. Such ensemble models generally improved model performance on various metrics, especially accuracy and AUC [59,60].

Proving the effectiveness of a deep learning model is also often challenging since it often functions like a black box. However, recent visualization methods, such as Grad-CAM [59,96] and back-propagation visualization [97], highlight salient areas of interest in images and may help to increase the trust and acceptance of both patients and clinicians in the AI model. For example, Jang et al. [34] described the use of Grad-CAM in their paper on chest radiographs and were able to present graphical illustrations of how the model made positive predictions based on various locations such as the humeral head, scapula, ribs, spine, and clavicle; it was noted that further work on visualization methods may help to improve the interpretability of AI models and improve clinician acceptance.

### 4.6. Future Directions in the Use of AI in Osteoporosis

There are numerous exciting opportunities for harnessing AI to predict osteoporosis from medical imaging. Osteoporosis may serve as an ideal stepping stone for the introduction of automated imaging systems to healthcare because the disease is highly prevalent with well-established benchmarks and reference standards in the form of DEXA but also not time-critical and amenable to further verification and follow-up. These automated platforms can then be integrated with other deep learning tools such as the detection of image quality, fractures, implants, and bony tumors that can help to reduce confounding factors for osteoporosis detection as well as serve as clinically relevant diagnostic tools in their own right.

Multimodality platforms may also serve as a useful direction for exploration. The use of CT assessment of osteoporosis is well established in the literature, with various tools showing excellent diagnostic performance [98,99]. MRI tools may also be useful: Zhao et al. proposed a fully automated radiomic screening pipeline for osteoporosis using a short lumbar mDIXON sequence for opportunistic screening, which could be performed in as short as 16 s [100]. A combined platform harnessing multiple modalities could yield higher accuracy with lower patient costs.

There is also great interest in the use of deep learning techniques to uncover further risk factors and predict fracture risk independent of BMD and FRAX [101]. For example, Yosibash et al. [102] described an autonomous algorithm combining autonomous finite element analysis and machine learning techniques for accurate prediction of future hip fracture risk assessment from CT scans of the abdomen and pelvis [102]. Further investigation and exploration of clinical integration of these methods is warranted.

Radiologists and clinicians must actively engage in the training and adoption of AI technologies and healthcare institutions must invest in infrastructure and education to support this transformative shift in medical imaging. Ultimately, a collaborative effort between clinicians, technology developers, and regulatory bodies is crucial to overcoming these challenges and realizing the full potential of AI in radiology.

## 5. Conclusions

This systematic review highlights the growing body of evidence that underscores the promise of harnessing artificial intelligence in radiographs for osteoporosis classification. Modern deep-learning technology allows for the automated analysis of substantial volumes of radiographic data, eliminating the need for labor-intensive manual segmentation and image analysis. In addition, the cost-effectiveness and accessibility of radiographs make them an ideal modality for large-scale population screening, particularly in settings where DEXA machines may be scarce. By leveraging the high diagnostic accuracy of deep learning models, there is a promising opportunity to enhance osteoporosis diagnosis and treatment initiation in these populations, thereby improving healthcare equity.

Several challenges must be addressed to fully realize the potential of AI-driven osteoporosis classification. Dataset collection is a major hurdle due to ethical concerns, data privacy issues, and biases. Standardizing methodologies and rigorously evaluating datasets are crucial for reliable and generalizable AI models across diverse populations. Moreover, ensuring robust algorithms capable of accurately distinguishing true bone mineral density alterations from other changes is essential given the technical quality of radiographs and the presence of confounding pathologies.

Looking ahead, collaboration between radiologists, clinicians, technology developers, and regulatory bodies is crucial to overcome the challenges associated with AI implementation and ensure patient-centric care. Future research endeavors should focus on addressing the challenges in technical application and clinical integration to facilitate future practical implementation of this technology.

## Data Availability

Not applicable.
